# Evaporation of a sessile droplet on a slope

**DOI:** 10.1038/s41598-019-55040-x

**Published:** 2019-12-24

**Authors:** Mitchel L. Timm, Esmaeil Dehdashti, Amir Jarrahi Darban, Hassan Masoud

**Affiliations:** 1 Department of Mechanical Engineering-Engineering Mechanics, Michigan Technological University, Houghton, Michigan 49931 USA; 2 Department of Physics, University of Nevada, Reno, Nevada 89557 USA

**Keywords:** Applied mathematics, Mechanical engineering, Fluid dynamics

## Abstract

We theoretically examine the drying of a stationary liquid droplet on an inclined surface. Both analytical and numerical approaches are considered, while assuming that the evaporation results from the purely diffusive transport of liquid vapor and that the contact line is a pinned circle. For the purposes of the analytical calculations, we suppose that the effect of gravity relative to the surface tension is weak, i.e. the Bond number (Bo) is small. Then, we express the shape of the drop and the vapor concentration field as perturbation expansions in terms of Bo. When the Bond number is zero, the droplet is unperturbed by the effect of gravity and takes the form of a spherical cap, for which the vapor concentration field is already known. Here, the Young-Laplace equation is solved analytically to calculate the first-order correction to the shape of the drop. Knowing the first-order perturbation to the drop geometry and the zeroth-order distribution of vapor concentration, we obtain the leading-order contribution of gravity to the rate of droplet evaporation by utilizing Green’s second identity. The analytical results are supplemented by numerical calculations, where the droplet shape is first determined by minimizing the Helmholtz free energy and then the evaporation rate is computed by solving Laplace’s equation for the vapor concentration field via a finite-volume method. Perhaps counter-intuitively, we find that even when the droplet deforms noticeably under the influence of gravity, the rate of evaporation remains almost unchanged, as if no gravitational effect is present. Furthermore, comparison between analytical and numerical calculations reveals that considering only the leading-order corrections to the shape of the droplet and vapor concentration distribution provides estimates that are valid well beyond their intended limit of very small Bo.

## Introduction

Liquid droplets are ubiquitous in daily life, whether it is a spilled beverage on a table or morning dew on the hood of a car. Everyone has seen these quasi-spherical liquid forms and how they evaporate over time if left alone. Perhaps even, when driving while it is raining, one may have made the observation that the shape of these drops change when on an inclined surface like a windshield. When on a slope, the seemingly round drops become more asymmetric with the majority of the volume shifted in the direction of the downward slope. A question that naturally arises is how does the droplet deformation caused by gravity affect the rate of evaporation. The answer to this question is not only of general interest from the fundamental point of view, but is also of great relevance to the real-world applications that involve the drying of sessile droplets. A large number of these applications deal with colloidal drops that leave behind a residue once they completely dry out. Common examples include ink-jet printing^1–6^ and fabricating ordered microelectronic structures via evaporative self-assembly^7–15^, where the time it takes for the deposit to form is a key design parameter.

To date, the majority of studies on the evaporation of sessile drops have focused on axisymmetric geometries on horizontal substrates^16–21^. This simplification, however useful, is limiting, because in many practical situations, the droplets may rest on an incline. Among a small number of investigations that considered asymmetric sessile droplets, Espín and Kumar^22^ and Du and Deegan^23^ numerically examined the drying of (and the resulting flow field inside) two-dimensional colloidal droplets on inclined substrates. Also, Sáenz *et al*.^24^ studied, both experimentally and numerically, the evaporation kinetics of non-axisymmetric drops placed on a flat surface and presented a general scaling law for the integrated evaporative flux. Lastly, Kim *et al*.^25^ experimentally measured the lifetime and evaporation dynamics of water droplets on slopes of various tilt angles. Overall, a close inspection of the literature indicates that our theoretical understanding of the effect of gravity on the evaporation of droplets sitting on tilted substrates is incomplete.

In an attempt to partially fill the aforementioned knowledge gap, here, we theoretically analyze the drying of a sessile droplet on a slope. The analyses involve perturbation calculations aided by Green’s second identity, and numerical simulations, which are intended to confirm and extend the analytical results. In both cases, the shape of the droplet and the evaporation rate are determined respectively. Throughout the study, it is assumed that the transport of the liquid vapor (from the surface of the droplet into an infinite ambient) is governed by Laplace’s equation and that the contact line is a pinned circle. Interestingly, we discover that even when the droplet geometry is significantly distorted by gravity, the rate at which the droplet loses mass changes only slightly. This and other findings of our study provide additional insights into the evaporation of droplets on oblique planes. In the following sections, we will first formulate the arising mathematical problems and describe their perturbation and numerical solutions. Then, we will present the results, discuss the implications, and provide a brief summary in the last section.

## Droplet Shape

**Figure 1:**
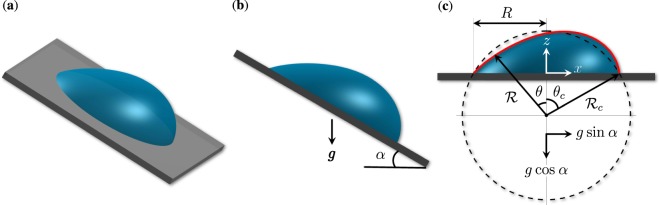
(**a**) A stationary droplet on an inclined surface. (**b**) Side view of panel (a), where the inclination angle is denoted by *α* and the arrow shows the direction of the gravity vector ***g***. (**c**) Rotated, by *α*, view of panel (b).

Consider a static droplet (at room conditions) on a flat plate at angle *α* to the horizontal plane and suppose that the gravity acts in the downward vertical direction (see Fig. 1). Before we can proceed to calculate the evaporation rate of the droplet (which is the primary objective of this study), it is necessary to first determine its shape. The equilibrium shape of the droplet is set by the force balance at the liquid-air interface, where the pressure difference across the interface is offset by the force of surface tension. Mathematically, the balance is expressed through the Young-Laplace equation

1 $$p-{p}_{atm}={\boldsymbol{\nabla }}\cdot {\boldsymbol{n}}=-\,2H,$$

where *p*, *p*_*atm*_, ***n***, and *H* denote the dimensionless hydrostatic pressure just below the interface, dimensionless atmospheric pressure, unit normal vector directed into the air, and dimensionless mean local curvature, respectively (see, e.g., ref. ^26^). Here and throughout the rest of the article, pressure and length are non-dimensionalized by $\gamma /{{\mathscr{R}}}_{c}$ and ${ {\mathcal R} }_{c}$, respectively, where *γ* is the surface tension and ${ {\mathcal R} }_{c}$ is a characteristic length of the droplet (see Fig. 1c). Equation (1) can also be derived from the minimum energy principle, which requires the equilibrium shape of the droplet to be the one that minimizes the Helmholtz free energy, subject to constant volume and other imposed constraints, if any (see, e.g., ref. ^26^).

Let $(x,y,z)$ be the components of a Cartesian coordinate system with the origin at the center of the contact line, as depicted in Fig. 1c. The hydrostatic pressure can then be expressed as

2 $$p={p}_{0}-{\rm{Bo}}\,z\,\cos \,\alpha +{\rm{Bo}}\,x\,\sin \,\alpha ,$$

where *p*_0_ is the reference pressure at the origin and ${\rm{Bo}}=\rho g{ {\mathcal R} }_{c}^{2}/\gamma$ is a dimensionless parameter called the Bond number, that measures the strength of gravity relative to surface tension. Here, $\rho$ and *g* represent the density of the liquid and the gravitational acceleration, respectively.

In the absence of gravity (i.e. ${\rm{Bo}}=0$), *p* does not vary along the interface (irrespective of the tilt angle *α*), and, therefore, $\Delta p=p-{p}_{atm}$ becomes a constant. Hence, the mean curvature *H* is constant, meaning that the droplet takes the form of a spherical cap with a contact angle that depends on the wetting properties of the substrate. When the effect of gravity compared to that of surface tension is not negligible (i.e. Bo is finite), the hydrostatic pressure no longer stays uniform and, as a result, the drop geometry deviates from a spherical cap. In general, Eq. (1) is challenging to solve analytically for arbitrary Bo in three dimensions. However, in situations where gravity is present, but weak (i.e. ${\rm{Bo}}\ll 1$), the shape of the drop can be treated as a slightly perturbed spherical cap (see, e.g., refs. ^27,28^). In this limit, the Young-Laplace equation can be solved via a regular perturbation expansion in terms of Bo^28^. In the following, we describe the derivation.

To simplify the calculations, we follow previous studies (see, e.g., refs. ^23,28,29^) and assume that the three-phase contact line is a pinned circle of radius *R* (see Fig. 1c). There is rich literature on the conditions under which a droplet sticks to or rolls off of an inclined substrate (see, e.g., refs. ^30–38^ and references therein). The presumed pinning is realized, for example, when the droplet is initially deposited on a horizontal substrate (where it forms a circular contact line) and then the substrate is gently tilted to an angle *α*, such that the contact angle at each point of the contact line is within the hysteresis range, whose lower and upper bounds are set by the receding and advancing contact angles, respectively. Considering that the unperturbed shape of the droplet is a spherical cap, it is more convenient to carry out the derivation in a spherical coordinate system whose components $(r,\theta ,\varphi )$ are related to the Cartesian coordinates as

3 $$x=r\,\sin \,\theta \,\cos \,\varphi ,\,\,y=r\,\sin \,\theta \,\sin \,\varphi ,\,\,z=r\,\cos \,\theta -\,\cos \,{\theta }_{c}.$$

Here, ${ {\mathcal R} }_{c}$ is the radius of curvature of the spherical cap and *θ*_*c*_ is the contact angle of the unperturbed droplet. By definition, these two parameters represent the values of *r* and *θ* at the contact line, respectively (see Fig. 1c). The dimensionless volume of the droplet *V* and the radius of the contact line *R* are related to *θ*_*c*_ via

4 $$V=\mathop{V}\limits^{ \sim }/{{\mathscr{R}}}_{c}^{3}=(\pi /3)(2+\,\cos \,{\theta }_{c}){(1-\cos {\theta }_{c})}^{2},\,R=\mathop{R}\limits^{ \sim }/{{\mathscr{R}}}_{c}=\,\sin \,{\theta }_{c},$$

where tilde overbars denote dimensional quantities. In $(r,\theta ,\varphi )$ coordinates, the free surface of the droplet can be described as the zero level set of

5 $$\Gamma (r,\theta ,\varphi ;{\rm{Bo}})=r- {\mathcal R} (\theta ,\varphi ;{\rm{Bo}}),$$

where ${\mathcal R}$ is the dimensionless shape function that we seek to determine for a given Bo. This function shall satisfy the condition ${\mathcal R} ({\theta }_{c},\varphi ;{\rm{Bo}})=1$ as well as the constraint that the volume enclosed between the free surface and the substrate be equal to *V*. Following Eq. (5), the right-hand side of Eq. (1) can be written as

6 $${\boldsymbol{\nabla }}\cdot {\boldsymbol{n}}=-\,2H={\boldsymbol{\nabla }}\cdot \frac{{\boldsymbol{\nabla }}\Gamma }{|{\boldsymbol{\nabla }}\Gamma |}.$$

Having set up the desired coordinate system, we now pose perturbation expansions as

7a $$ {\mathcal R} ={ {\mathcal R} }^{(0)}+{\rm{Bo}}\,{ {\mathcal R} }^{(1)}+{\mathscr{O}}({{\rm{Bo}}}^{2}),$$

7b $$H={H}^{(0)}+{\rm{Bo}}\,{H}^{(1)}+{\mathscr{O}}({{\rm{Bo}}}^{2}),$$

7c $${p}_{0}={p}_{0}^{(0)}+{\rm{Bo}}\,{p}_{0}^{(1)}+{\mathscr{O}}({{\rm{Bo}}}^{2}),$$

which, upon substitution into Eq. (1) and applying the pinned contact line condition, yield

8a $${p}_{0}^{(0)}={p}_{atm}-2{H}^{(0)}\,{\rm{w}}{\rm{i}}{\rm{t}}{\rm{h}}\,{{\mathscr{R}}}^{(0)}({\theta }_{c},\varphi )=1,$$

8b $$\begin{array}{c}{p}_{0}^{(1)}=({{\mathscr{R}}}^{(0)}\,\cos \,\theta -\,\cos \,{\theta }_{c})\,\cos \,\alpha -\,{{\mathscr{R}}}^{(0)}\,\sin \,\theta \,\cos \,\varphi \,\sin \,\alpha -2{H}^{(1)}\,{\rm{w}}{\rm{i}}{\rm{t}}{\rm{h}}\,{{\mathscr{R}}}^{(1)}({\theta }_{c},\varphi )=0.\end{array}$$

Note that the reference pressure is not known *a priori* and is calculated as a part of the solution.

As discussed earlier, at the zeroth order, we have

9 $${ {\mathcal R} }^{(0)}=1,\,{H}^{(0)}=-\,1,\,{p}_{0}^{(0)}={p}_{atm}+2.$$

Replacing Eq. (7a) for ${\mathcal R}$ in Eq. (5), while accounting for Eq. (9), and then substituting the result into Eq. (6), we obtain

10 $${H}^{(1)}=\frac{1}{2}(\frac{{\partial }^{2}{ {\mathcal R} }^{(1)}}{\partial {\theta }^{2}}+\frac{1}{{\sin }^{2}\,\theta }\frac{{\partial }^{2}{ {\mathcal R} }^{(1)}}{\partial {\varphi }^{2}}+\frac{1}{\tan \,\theta }\frac{\partial { {\mathcal R} }^{(1)}}{\partial \theta }+2{ {\mathcal R} }^{(1)})$$

for the first-order correction to the mean curvature of the interface. Equations (8b) and (10) together constitute a partial differential equation (PDE) for ${ {\mathcal R} }^{(1)}$. The linearity and structure of this PDE suggest a superposition solution in the form of

11 $${ {\mathcal R} }^{(1)}(\theta ,\varphi )=\Re (\theta )\,\cos \,\alpha + {\mathcal R} (\theta )\,\cos \,\varphi \,\sin \,\alpha ,$$

where $\Re$ and ${\mathcal R}$ represent the axisymmetric and non-axisymmetric deformation of the droplet, respectively, and satisfy the following ordinary differential equations:

12 $${{\rm{\Re }}}^{{\rm{^{\prime} }}{\rm{^{\prime} }}}+\frac{{{\rm{\Re }}}^{{\rm{^{\prime} }}}}{\tan \,\theta }+2{\rm{\Re }}-\,\cos \,\theta +\,\cos \,{\theta }_{c}+\frac{{p}_{0}^{(1)}}{\cos \,\alpha }=0\,{\rm{w}}{\rm{i}}{\rm{t}}{\rm{h}}\,{{\rm{\Re }}}^{{\rm{^{\prime} }}}(0)={\rm{\Re }}({\theta }_{c})=0,$$

13 $${ {\mathcal R} }^{{\rm{^{\prime} }}{\rm{^{\prime} }}}+\frac{{ {\mathcal R} }^{{\rm{^{\prime} }}}}{\tan \,\theta }+(2-\frac{1}{{\sin }^{2}\,\theta }) {\mathcal R} +\,\sin \,\theta =0\,{\rm{w}}{\rm{i}}{\rm{t}}{\rm{h}}\, {\mathcal R} (0)= {\mathcal R} ({\theta }_{c})=0.$$

The boundary conditions at $\theta =0$ ensure that the interface is continuous and smooth. Also, ${p}_{0}^{(1)}$ is included in the equation for $\Re$ because it is invariant to changes in the tilt angle from *α* to −*α*, which indicates that its value is proportional to cos *α*.

The exact solutions of Eqs. (12) and (13) are, respectively,

14 $$\begin{array}{rcl}\Re (\theta ) & = & \frac{1}{6}[(3\,\cos \,{\theta }_{c}+\frac{3{p}_{0}^{(1)}}{\cos \,\alpha }-2)(\frac{\cos \,\theta }{\cos \,{\theta }_{c}}-1)-2\,\cos \,\theta \,\mathrm{ln}\,(\frac{1+\,\cos \,\theta }{1+\,\cos \,{\theta }_{c}})]\\  & = & \frac{1}{6}\{\cos \,\theta \,[1-2\,\mathrm{ln}\,(\frac{1+\,\cos \,\theta }{1+\,\cos \,{\theta }_{c}})]-\,\cos \,{\theta }_{c}\},\end{array}$$

15 $$ {\mathcal R} (\theta )=\frac{1}{3}\{\sin \,\theta \,[\mathrm{ln}(\frac{1+\,\cos \,\theta }{1+\,\cos \,{\theta }_{c}})-\,\tan \,\frac{{\theta }_{c}}{2}\,\cot \,{\theta }_{c}]+\,\tan \,\frac{\theta }{2}\,\cos \,\theta \},$$

where

16 $${p}_{0}^{(1)}=\frac{2}{3}(1-\,\cos \,{\theta }_{c})\,\cos \,\alpha .$$

The first-order correction to the reference pressure is calculated by enforcing that the perturbations due to ${ {\mathcal R} }^{(1)}$ do not alter the volume of the droplet, which can be written as

17 $$\begin{array}{rcl}V & = & {\int }_{0}^{2\pi }\,{\int }_{0}^{{\theta }_{c}}\,{\int }_{0}^{ {\mathcal R} }\,{r}^{2}\,\sin \,\theta \,{\rm{d}}r\,{\rm{d}}\theta \,{\rm{d}}\varphi -\frac{\pi }{3}\,\cos \,{\theta }_{c}(1-{\cos }^{2}\,{\theta }_{c})\\  & = & {\int }_{0}^{2\pi }\,{\int }_{0}^{{\theta }_{c}}\,(\frac{{ {\mathcal R} }^{3}}{3})\,\sin \,\theta \,{\rm{d}}\theta \,{\rm{d}}\varphi -\frac{\pi }{3}\,\cos \,{\theta }_{c}(1-{\cos }^{2}\,{\theta }_{c})\\  & = & \frac{\pi }{3}(2+\,\cos \,{\theta }_{c}){(1-\cos {\theta }_{c})}^{2}+{\rm{Bo}}\,{\int }_{0}^{2\pi }\,{\int }_{0}^{{\theta }_{c}}\,{ {\mathcal R} }^{(1)}\,\sin \,\theta \,{\rm{d}}\theta \,{\rm{d}}\varphi +{\mathscr{O}}({{\rm{Bo}}}^{2}).\end{array}$$

Thus, we have required

18 $${\int }_{0}^{2\pi }\,{\int }_{0}^{{\theta }_{c}}\,{ {\mathcal R} }^{(1)}\,\sin \,\theta \,{\rm{d}}\theta \,{\rm{d}}\varphi ={\int }_{0}^{2\pi }\,{\int }_{0}^{{\theta }_{c}}\,[\Re (\theta )\,\cos \,\alpha + {\mathcal R} (\theta )\,\cos \,\varphi \,\sin \,\alpha ]\,\sin \,\theta \,{\rm{d}}\theta \,{\rm{d}}\varphi =0,$$

which reduces to

19 $${\int }_{0}^{{\theta }_{c}}\,\Re (\theta )\,\sin \,\theta \,{\rm{d}}\theta =0.$$

Finally, we note that, our results are in complete agreement with those obtained by De Coninck *et al*.^28^, who carried out their derivation (with some additional steps) in a cylindrical coordinate system centered at $(x=0,y=0,z=0)$, and handled overhangs and ${\theta }_{c} > \pi /2$ by a transformation to spherical coordinates.

The above perturbation solution is expected to be accurate for small Bond numbers. To cover a wider range of Bo, one could continue solving for higher-order corrections analytically, which is mathematically involved. Here, instead, we resort to numerical simulation. As mentioned earlier, the shape of the droplet can be determined alternatively by minimizing the Helmholtz free energy of the system, given the constraints of constant volume and pinned circular contact line. In fact, this approach is more amenable to numerical computation. We use the *Surface Evolver*^39,40^ to numerically solve for the shape of the droplet, with no restriction on Bo as long as the results are physically meaningful. The *Surface Evolver* is a well-established computer program that minimizes the energy of a surface (which can take various forms) subject to constraints. Details of the implementation can be found in refs. ^39–41^. For each simulation, extra iteration steps and additional mesh refinement are implemented to ensure the convergence of the solution to the final geometry. Below, we discuss our findings concerning the shape of the droplet for various values of Bo, *θ*_*c*_, and *α*.

**Figure 2:**
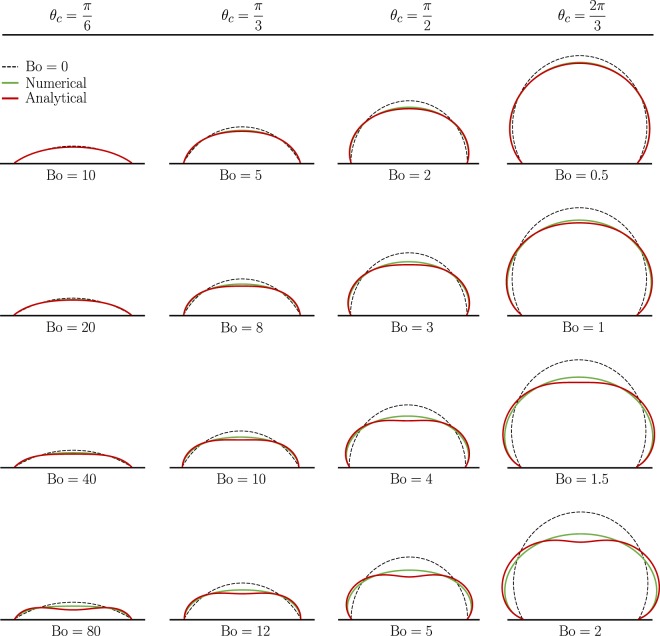
Center-line profiles of sessile drops sitting on a horizontal substrate for different values of Bo and *θ*_*c*_.

**Figure 3:**
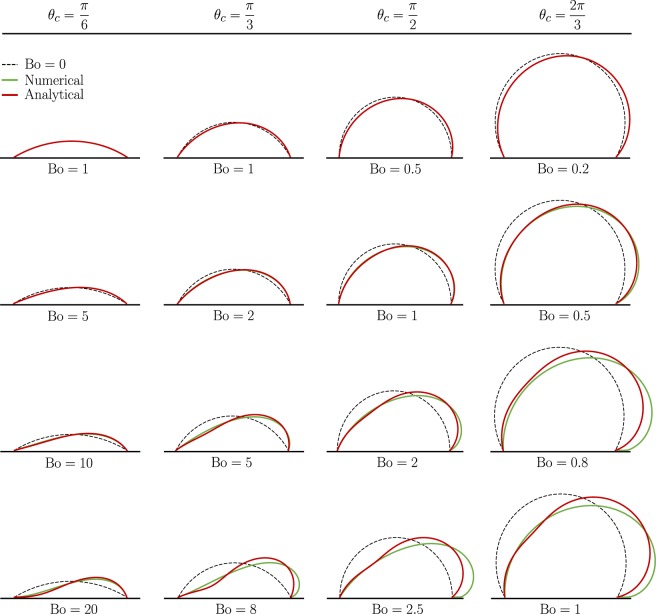
Center-line profiles of sessile drops sitting on a tilted substrate with an inclination angle $\alpha =\pi /6$ for different values of Bo and *θ*_*c*_.

Figures 2 and 3 present the results of numerical calculations (green solid lines) along with the predictions of the perturbation theory (red solid lines). To facilitate the comparison, two-dimensional profiles are shown along the symmetry plane of the droplet, where the largest deviations are manifested. Unperturbed profiles are also depicted as black dashed lines for reference. Each column in Figs. 2 and 3 refers to a different *θ*_*c*_, and each comparison plot corresponds to the Bo displayed beneath it. The Bond number generally increases in each successive row, allowing for easy visualization of trends. Note that our results can be readily contrasted against experimental data. For instance, if the volume $\tilde{V}$ and the contact angle of the unperturbed droplet *θ*_*c*_ are known, then one can use Eq. (4) to calculate ${ {\mathcal R} }_{c}$, from which the Bond number is determined (given that $\rho$ and *γ* are known).

First, we consider how the Bond number affects the shape of the drop before introducing any inclination to the substrate, i.e. we set $\alpha =0$. The results are illustrated in Fig. 2. In-line with intuition, we see that, generally, gravity tends to flatten the droplet, resulting in an increase in the contact angle and a decrease in the maximum height at a fixed contact radius. More importantly, we see very good agreements between analytical and numerical results that, in many cases, extend well beyond the expected limit of ${\rm{Bo}}\ll 1$. In all cases, the discrepancy between theoretical predictions and simulations grows as Bo increases. Also, at a given Bond number, the difference is larger for higher *θ*_*c*_ that corresponds to greater maximum height. In other words, had we chosen the maximum height of the unperturbed droplet as the characteristic length in the definition of Bo, we would have seen comparable levels of discrepancy for similar values of Bo.

Next, we examine the effect of the inclination angle by increasing *α* to *π*/6 in Fig. 3. As it can be seen in this figure, there exist many of the same features as in the case where $\alpha =0$, including favorable agreements between perturbation and numerical solutions over a wide range of Bo. However, here, in addition to being compressed down, the droplets are tilted to the right and are no longer axisymmetric. Furthermore, the Bond number at which deviations between theory and simulation start to develop decreases as the angle of the slope is increased. For instance, at $\alpha =0$ and ${\theta }_{c}=\pi /2$, major deviations do not occur until ${\rm{Bo}}\approx 3$, whereas when $\alpha =\pi /6$ the critical limit is closer to ${\rm{Bo}}=2$. Very similar observations are made when *α* is further elevated to *π*/3 and *π*/2 (see Supplementary Figs. S1 and S2).

## Droplet Evaporation

Now that we have determined the equilibrium shape of the droplet, we are in a position to answer the question we posed in the introduction, which focused on the effect of droplet deformation on how fast the drop evaporates. The rate at which the droplet loses mass is obtained by integrating the flux of the liquid vapor concentration in air over the free surface of the drop (denoted *S*_*d*_), i.e.

20 $$J=-\,\mathop{\int }\limits_{{S}_{d}}\,{\boldsymbol{n}}\cdot {\boldsymbol{\nabla }}c\,{\rm{d}}S,$$

where all the quantities are dimensionless. Specifically, the vapor concentration field *c* and total evaporation rate *J* are non-dimensionalized by ${c}_{s}-{c}_{\infty }$ and $D{ {\mathcal R} }_{c}({c}_{s}-{c}_{\infty })$, respectively. Here, *c*_*s*_ is the vapor concentration at *S*_*d*_ (saturation value), ${c}_{\infty }$ is the far-field concentration, and *D* is the coefficient of binary diffusion of the vapor in air.

We assume that the transport of the liquid vapor in the surrounding quiescent air is dominated by diffusion. This assumption is valid when the time scale for the diffusion of the vapor concentration ${\tau }_{d}$ is much smaller than the total evaporation time of the droplet *t*_*f*_. For millimeter-sized water droplets drying in still air at room conditions, the ratio ${\tau }_{d}/{t}_{f}$ is very small, of the order of 10^−5^ (see, e.g., refs. ^16,42^). Additionally, for slowly evaporating drops under (nearly) isothermal conditions, the advective vapor transport by the Stefan and buoyancy-driven flows can be neglected^18,43^. Hence, defining $\phi =c-{c}_{\infty }$/$({c}_{s}-{c}_{\infty })$, we have

21 $${{\rm{\nabla }}}^{2}\phi =0\,{\rm{w}}{\rm{i}}{\rm{t}}{\rm{h}}\,\phi =1\,{\rm{a}}{\rm{t}}\,{S}_{d},\,{\boldsymbol{n}}\cdot {\boldsymbol{\nabla }}\phi =0\,{\rm{a}}{\rm{t}}\,{S}_{s},\,{\rm{a}}{\rm{n}}{\rm{d}}\,\phi \to 0\,{\rm{a}}{\rm{s}}\,r\to {\rm{\infty }},$$

where *S*_*s*_ represents the surface of the substrate. In what follows, we derive a closed-form expression for *J* in the limit of small Bo based on the perturbation solution obtained in the previous section.

Given that *S*_*d*_ is described by $r= {\mathcal R} =1+{\rm{Bo}}\,{ {\mathcal R} }^{(1)}+{\mathscr{O}}({{\rm{Bo}}}^{2})$, it is only natural to express the relative concentration field $\phi$ as

22 $$\phi ={\phi }^{(0)}+{\rm{Bo}}\,{\phi }^{(1)}+{\mathscr{O}}({{\rm{Bo}}}^{2}),$$

which, upon substitution into Eq. (21), yields

23a $${{\rm{\nabla }}}^{2}{\phi }^{(0)}=0\,{\rm{w}}{\rm{i}}{\rm{t}}{\rm{h}}\,{\boldsymbol{n}}\cdot {\boldsymbol{\nabla }}{\phi }^{(0)}=0\,{\rm{a}}{\rm{t}}\,{S}_{s}\,{\rm{a}}{\rm{n}}{\rm{d}}\,{\phi }^{(0)}\to 0\,{\rm{a}}{\rm{s}}\,r\to {\rm{\infty }},$$

23b $${{\rm{\nabla }}}^{2}{\phi }^{(1)}=0\,\text{with}\,{\boldsymbol{n}}\cdot {\boldsymbol{\nabla }}{\phi }^{(1)}=0\,{\rm{a}}{\rm{t}}\,{S}_{s}\,{\rm{a}}{\rm{n}}{\rm{d}}\,{\phi }^{(1)}\to 0\,{\rm{a}}{\rm{s}}\,r\to {\rm{\infty }},$$

where the boundary conditions at *S*_*d*_ are omitted since an extra step is required to derive them. Consider a Taylor series expansion of $\phi$ about $r=1$ as

24 $$\phi (r,\theta ,\varphi )=\phi (1,\theta ,\varphi )+(r-1){\frac{\partial \phi }{\partial r}|}_{r=1}+\ldots ,$$

and apply the boundary condition $\phi ( {\mathcal R} ,\theta ,\varphi )=1$, while replacing Eq. (22) for $\varphi$, to arrive at

25 $$\begin{array}{c}\phi (r={\mathscr{R}},\theta ,\varphi )={\varphi* }^{(0)}(1,\theta ,\varphi )+{\rm{B}}{\rm{o}}\,[{\varphi* }^{(1)}(1,\theta ,\varphi )+{{\mathscr{R}}}^{(1)}{\frac{{\rm{\partial }}{\varphi* }^{(0)}}{{\rm{\partial }}r}|}_{r=1}]+{\mathscr{O}}({{\rm{B}}{\rm{o}}}^{2})=1.\end{array}$$

Requiring this equation to hold for each order of Bo, we find

26 $${\varphi* }^{(0)}(1,\theta ,\varphi )=1\,{\rm{a}}{\rm{n}}{\rm{d}}\,{\varphi* }^{(1)}(1,\theta ,\varphi )=-\,{{\mathscr{R}}}^{(1)}{\frac{{\rm{\partial }}{\varphi* }^{(0)}}{{\rm{\partial }}r}|}_{r=1},$$

which complete the boundary value problems described by Eqs. (23a) and (23b). Here, we have essentially converted the original boundary condition at *S*_*d*_ to a set of boundary conditions at the surface of the spherical cap $r=1$, which we refer to hereafter as *S*_0_.

To proceed, we also expand *J* in terms of Bo as

27 $$J={J}^{(0)}+{\rm{Bo}}\,{J}^{(1)}+{\mathscr{O}}({{\rm{Bo}}}^{2}),$$

where

28 $${J}^{(i)}=-\mathop{\int }\limits_{{S}_{d}}\,{\boldsymbol{n}}\cdot {\boldsymbol{\nabla }}{\varphi* }^{(i)}\,{\rm{d}}S=-\mathop{\int }\limits_{{S}_{0}}\,{\boldsymbol{n}}\cdot {\boldsymbol{\nabla }}{\varphi* }^{(i)}\,{\rm{d}}S\,{\rm{w}}{\rm{i}}{\rm{t}}{\rm{h}}\,i=0,1.$$

The fact that *S*_*d*_ can be replaced with *S*_0_ in the above surface integral directly results from having ${\nabla }^{2}{\varphi* }^{(i)}=0$, everywhere in the domain including in the volume enclosed between *S*_0_ and *S*_*d*_. Laplace’s Eq. (23a) for ${\varphi* }^{(0)}$, with the specified Dirichlet boundary conditions at $r=1$ (see Eq. (26)) and $r\to \infty$, can be solved exactly in a toroidal coordinate system that fits the boundary of the droplet using a special form of the method of separation of variables. Details of the derivation is available in ref. ^44^. Thus, ${\varphi* }^{(0)}$ is already known, and so are its normal gradient at *S*_0_ and its corresponding surface integral, which can be written in the forms

29a $$\begin{array}{rcl}{{\boldsymbol{n}}\cdot {\boldsymbol{\nabla }}{\varphi* }^{(0)}|}_{{S}_{0}}={\frac{\partial {\varphi* }^{(0)}}{\partial r}|}_{r=1} & = & -\frac{1}{2}-\frac{\sqrt{2}\,{\sin }^{2}\,{\theta }_{c}}{{(\cos \theta -\cos {\theta }_{c})}^{3/2}}\\  &  & \times \,{\int }_{0}^{\infty }\frac{\cosh \,{\theta }_{c}\tau }{\cosh \,\pi \tau }\,\tanh \,[(\pi -{\theta }_{c})\tau ]\\  &  & \times \,{P}_{-1/2+i\tau }(\frac{1-\,\cos \,\theta \,\cos \,{\theta }_{c}}{\cos \,\theta -\,\cos \,{\theta }_{c}})\tau \,{\rm{d}}\tau ,\end{array}$$

29b $$\begin{array}{ccc}{J}^{(0)} & = & -\,{\int }_{0}^{2\pi }\,{\int }_{0}^{{\theta }_{c}}\,{\frac{{\rm{\partial }}{\varphi*! }^{(0)}}{{\rm{\partial }}r}|}_{r=1}\,\sin \,\theta \,{\rm{d}}\theta \,{\rm{d}}\varphi \\  & = & -2\pi \,{\int }_{0}^{{\theta }_{c}}\,{\frac{{\rm{\partial }}\varphi }{{\rm{\partial }}r}|}_{r=1}\,\sin \,\theta \,{\rm{d}}\theta \\  & = & \pi \,\sin \,{\theta }_{c}\{\frac{\sin \,{\theta }_{c}}{1+\,\cos \,{\theta }_{c}}+4\,{\int }_{0}^{{\rm{\infty }}}\,\frac{1+\,\cosh \,2{\theta }_{c}\tau }{\sinh \,2\pi \tau }\,\tanh \,[(\pi -{\theta }_{c})\,\tau ]\,{\rm{d}}\tau \},\end{array}$$

where ${P}_{-1/2+i\tau }$ is the the conical function of the first kind, that can be evaluated via

30 $${P}_{\nu }(\zeta )=\frac{1}{\pi }\,{\int }_{0}^{\pi }\,{(\zeta +\cos \eta \sqrt{{\zeta }^{2}-1})}^{-(\nu +1)}\,{\rm{d}}\eta .$$

Equations (29a) and (29b) are indeed the commonly reported local and total rates of evaporation from spherical-cap drops, respectively (see, e.g., ref. ^45^). This ensures that we recover the existing exact solution for the evaporation rate when ${\rm{Bo}}=0$.

So, we are left to determine *J*^(1)^. If we were to follow the conventional approach, we would first solve Eq. (23b) for ${\varphi }^{(1)}$ and then integrate the associated flux over *S*_0_ to obtain *J*^(1)^. However, we take an alternative approach – that bypasses the tedious task of solving for ${\varphi }^{(1)}$ – and directly calculate *J*^(1)^. Consider multiplying Laplace’s Eq. (23b) by ${\varphi }^{(0)}$ and Laplace’s Eq. (23a) by ${\varphi }^{(1)}$, and then subtracting to reach

31 $${\varphi }^{(0)}{\nabla }^{2}{\varphi }^{(1)}-{\varphi }^{(1)}{\nabla }^{2}{\varphi }^{(0)}=0.$$

After adding and subtracting ${\boldsymbol{\nabla }}{\varphi }^{(0)}\cdot {\boldsymbol{\nabla }}{\varphi }^{(1)}$ and rearranging, this equation can be rewritten as

32 $${\boldsymbol{\nabla }}\cdot ({\varphi }^{(0)}{\boldsymbol{\nabla }}{\varphi }^{(1)})={\boldsymbol{\nabla }}\cdot ({\varphi }^{(1)}{\boldsymbol{\nabla }}{\varphi }^{(0)}).$$

We now integrate Eq. (32) over the volume external to the drop and use the divergence theorem to obtain

33 $$\mathop{\int }\limits_{S}\,{\varphi }^{(0)}\,{\boldsymbol{n}}\cdot {\boldsymbol{\nabla }}{\varphi }^{(1)}\,{\rm{d}}S=\mathop{\int }\limits_{S}\,{\varphi }^{(1)}\,{\boldsymbol{n}}\cdot {\boldsymbol{\nabla }}{\varphi }^{(0)}\,{\rm{d}}S,$$

where *S* denotes all the surfaces bounding the distribution domain of ${\varphi }^{(0)}$ and ${\varphi }^{(1)}$, i.e. $S={S}_{0}+{S}_{s}+{S}_{\infty }$, with ${S}_{\infty }$ representing a bounding surface at large distances. The above result is known as Green’s second identity and is the scalar version of the reciprocal theorem often used in solid and fluid mechanics (see, e.g., ref. ^46^). It is also a special case of the reciprocal theorem developed by Vandadi *et al*.^47^ for convection heat and mass transfer from particles in Stokes and potential flows.

Contributions from ${S}_{\infty }$ to Eq. (33) vanish since both ${\varphi }^{(0)}$ and ${\varphi }^{(1)}$ decay at least as fast as 1/*r* as $r\to \infty$. Integrals over the substrate do not contribute either as ${\boldsymbol{n}}\cdot {\boldsymbol{\nabla }}{\varphi }^{(i)}=0$ at *S*_*s*_. Hence, applying Eq. (26), we find

34 $${J}^{(1)}=-\,\mathop{\int }\limits_{{S}_{0}}\,{\boldsymbol{n}}\cdot {\boldsymbol{\nabla }}{\varphi }^{(1)}\,{\rm{d}}S={\int }_{0}^{2\pi }\,{\int }_{0}^{{\theta }_{c}}\,{ {\mathcal R} }^{(1)}{({\frac{\partial {\varphi }^{(0)}}{\partial r}|}_{r=1})}^{2}\,\sin \,\theta \,{\rm{d}}\theta \,{\rm{d}}\varphi ,$$

which, after incorporating Eq. (11) for ${ {\mathcal R} }^{(1)}$, simplifies to

35 $${J}^{(1)}=2\pi \,\cos \,\alpha \,{\int }_{0}^{{\theta }_{c}}\,\Re (\theta ){({\frac{\partial {\varphi }^{(0)}}{\partial r}|}_{r=1})}^{2}\,\sin \,\theta \,{\rm{d}}\theta .$$

Note that the integral involving ${\mathcal R}$ is zero because ${\int }_{0}^{2\pi }\,\cos \,\varphi \,{\rm{d}}\varphi =0$.

To complement our analytical endeavor, we also solve the boundary value problem described by Eq. (21) numerically and compute the rate of evaporation for the geometries calculated by the *Surface Evolver* (see Figs. 2 and 3, and Supplementary Figs. S1 and S2). Using these geometries allows us to conduct simulations for higher Bo, since the analytically obtained shapes become less accurate with increasing Bo. By running simulations for higher Bo (beyond the ${\rm{Bo}}\ll 1$ condition), we can find the true limit for which our analytical formula for the rate of evaporation is valid.

**Figure 4:**
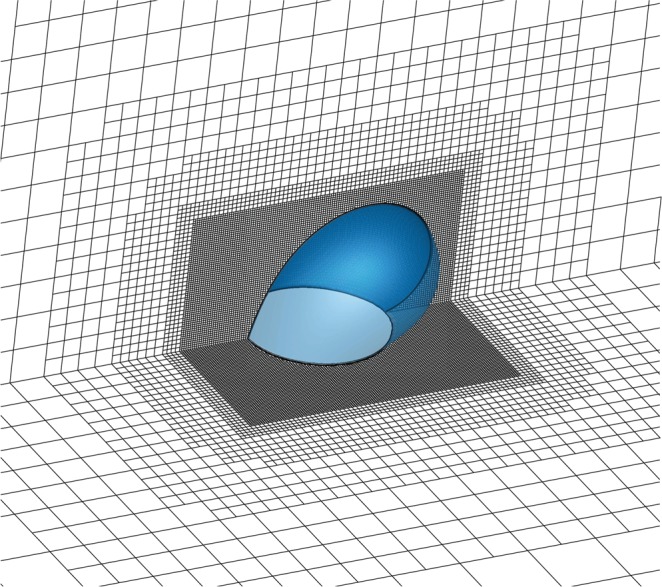
Example of a multi-block grid used for numerically solving Laplace’s equation outside a sessile droplet (shown in blue). For clarity, only surface meshes on the symmetry plane and substrate are shown. The grid is generated by the *snappyHexMesh* utility of *OpenFOAM*^48^, and coarsens gradually as the distance from the free surface of the drop increases.

We use a finite-volume method as implemented in *OpenFOAM* (see, e.g., ref. ^48^). The Laplacian is discretized via the second-order linear Gaussian integration, and the corrected scheme, with the number of correction set to two, is employed to calculate surface normal gradients. The latter accounts for the non-orthogonality of the mesh generated by the *snappyHexMesh* utility, especially at the surface of the droplet (see Fig. 4). The outer boundary at infinity is modeled by a hemisphere of radius 100 *R*, whose center coincides with the center of the contact line circle. Given our large domain size, we use a multi-block mesh that is refined in the vicinity of the droplet to reduce the overall number of computational cells, while accurately resolving the gradients where they matter the most (see Fig. 4). The smallest and largest grid spacings used are approximately 0.008*R* and 8*R*, respectively.

## Results and Discussion

We begin with analyzing the results of the perturbation calculations. There are a couple of points that can be deduced right away from the final form of *J*^(1)^ (see Eq. (35)). First, to the leading order in Bo, the asymmetric deformation of the droplet, represented by ${\mathcal R}$, does not contribute to the total rate of evaporation, regardless of the inclination angle *α*. Second, the first-order correction to the evaporation rate is proportional to cos *α*, which means that, generally, for a given *θ*_*c*_, *J*^(1)^ decreases as *α* increases and it vanishes for $\alpha =\pi /2$. Thus, when the substrate is parallel to the direction of gravity, the deviation of *J* from *J*^(0)^ is of ${\mathscr{O}}({{\rm{Bo}}}^{2})$.

**Figure 5:**
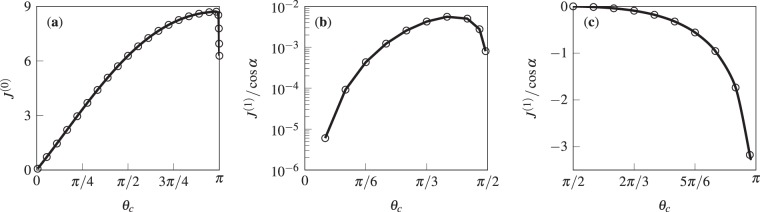
Plots of (**a**) *J*^(0)^ and (**b** and **c**) ${J}^{(1)}/\cos \,\alpha$ versus *θ*_*c*_, calculated based on Eqs. (29b) and (35), respectively.

Additional information can be inferred by scrutinizing the values of *J*^(0)^ and ${J}^{(1)}/\cos \,\alpha$ for various *θ*_*c*_ (see Fig. 5). For instance, we notice that the zeroth-order evaporation rate increases almost linearly from 0 to 2*π* as *θ*_*c*_ changes from 0 to *π*/2. As *θ*_*c*_ further increases, the rise in *J*^(0)^ continues with a lower slope until it reaches a temporary plateau, after which it suddenly decays to 2*π* as *θ*_*c*_ approaches *π*. The behavior of *J*^(0)^ at vanishing contact angles stems from the fact that the rate of evaporation is non-dimensionalized by $D{ {\mathcal R} }_{c}({c}_{s}-{c}_{\infty })$, and that, for a given non-zero contact radius, ${ {\mathcal R} }_{c}\to \infty$ as ${\theta }_{c}\to 0$. We also find that the ${\mathscr{O}}({\rm{Bo}})$ correction to the rate of evaporation is positive for $0 < {\theta }_{c} < \pi /2$, negative for $\pi /2 < {\theta }_{c} < \pi$, and zero for ${\theta }_{c}=\pi /2$. The latter suggests that $J-{J}^{(0)}$ varies to the leading order with Bo^2^ when the unperturbed geometry is a hemisphere. More importantly, inspection of the ratio *J*^(1)^/*J*^(0)^ for droplets of different (dimensionless) volume reveals that the variation of *J* due to the deformation of the droplet is minor (a few percent or less) even when the change in the geometry is quite pronounced. Consider, as extreme examples, the droplets in the last row of Fig. 2. According to our perturbation theory, the deformation-induced change in the rate of evaporation is below 3% for all cases.

**Figure 6:**
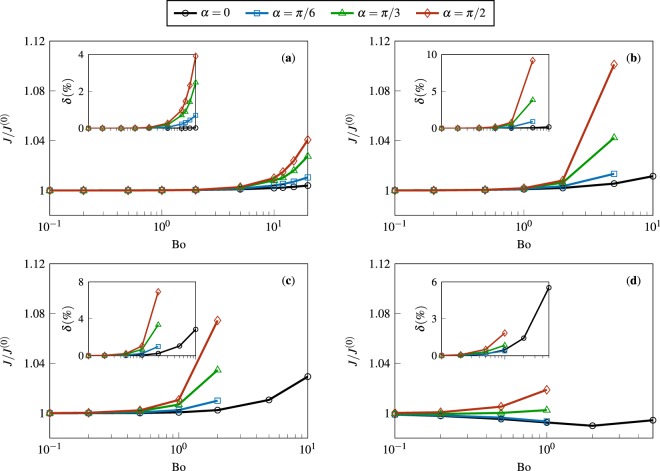
The normalized rate of evaporation *J*/*J*^(0)^, calculated numerically via *OpenFOAM*, as a function of the Bond number Bo for various substrate inclination angles *α*. Panels (a–d) correspond to ${\theta }_{c}=\pi /6,\,\pi /3,\,\pi /2,\,{\rm{and}}\,2\pi /3$, respectively. The insets show the percent difference *δ* between the results presented in (a)–(d) and the analytical predictions (see Fig. 5). In panels (b–d), the high-Bond-number data that correspond to unrealistic droplet shapes are not shown.

Our rather counter-intuitive analytical predictions are corroborated by numerical simulations; the results of which are presented in Fig. 6 in the form of normalized rate of evaporation *J*/*J*^(0)^ as a function of Bond number for *α* and *θ*_*c*_ studied in Figs. 2 and 3, and Supplementary Figs. S1 and S2. To quantitatively assess the accuracy of the perturbation theory, the percent difference between analytical and numerical results for *J*/*J*^(0)^ are shown in the insets. As expected, a very close match is seen for ${\rm{Bo}}\ll 1$. Remarkably, we also observe a small difference when Bo is ${\mathscr{O}}(1)$. For larger Bond numbers, the next order corrections, i.e. ${\mathscr{O}}({{\rm{Bo}}}^{2})$ and ${\mathscr{O}}({{\rm{Bo}}}^{3})$, become dominant and thus, a mismatch begins to develop. Nevertheless, the percent difference remains less than 10% for all the Bo considered. Overall, our calculations indicate decisively that the total evaporation rate of sessile droplets, unlike their geometry, is a very weak function of gravity.

**Figure 7:**
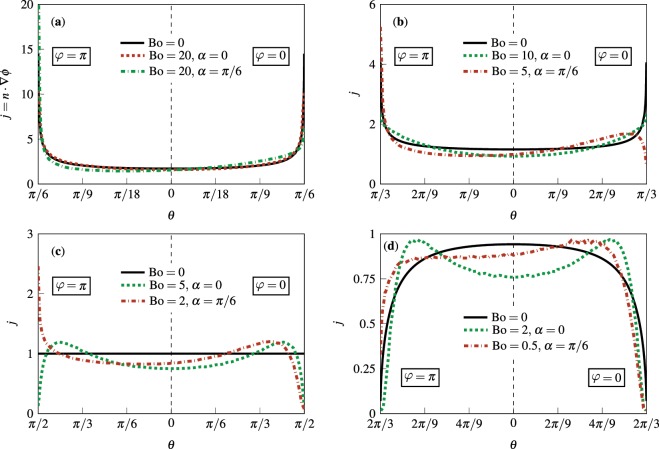
The normalized evaporative flux $j={\boldsymbol{n}}\cdot {\boldsymbol{\nabla }}\varphi$ along the droplet’s center line for representative combinations of Bond number Bo and substrate inclination angle *α*. Solid black lines represent exact analytical results for ${\rm{Bo}}=0$ (see Eq. (29a)) whereas dotted green and dash-dotted red lines show the results of numerical calculations via *OpenFOAM*. Panels (a–d) correspond to ${\theta }_{c}=\pi /6,\,\pi /3,\,\pi /2,\,{\rm{and}}\,2\pi /3$, respectively.

To better understand this phenomenon, we plot the variation of the evaporative flux $j={\boldsymbol{n}}\cdot {\boldsymbol{\nabla }}\varphi$ on *S*_*d*_ along the symmetry plane of the droplet for several representative combinations of Bo and *α* in Fig. 7. For axisymmetrically deformed droplets (corresponding to $\alpha =0$), we see that, as *θ* increases from zero, the flux profiles start below the base curves for ${\rm{Bo}}=0$ (corresponding to undeformed spherical-cap drops), then they cross the curves twice (the first time from below and the second time from above), and finally either blow up or decay to zero depending on their contact angle. Thus, relative to the corresponding Bo case, the local flux is higher between the two crosses and lower elsewhere, while the total evaporation rate is nearly the same. The plots of *j* on the downhill ($\varphi =0$) side of asymmetrically deformed droplets (corresponding to $\alpha =\pi /6$) behave qualitatively similar to those for $\alpha =0$. However, the plots on the uphill ($\varphi =\pi$) side follow a different trend, in that they cross the base curves once. Therefore, on the uphill side, the evaporative flux, compared to the corresponding ${\rm{Bo}}=0$ case, is lower before the cross-over point and higher thereafter. Nevertheless, the integral of *j* over *S*_*d*_ is, again, within a few percent of the base case.

As stated in §Introduction, there exist, at least, two recent studies that considered the evaporation of asymmetric three-dimensional sessile droplets. Hence, our work would be incomplete without discussing these investigations in the context of our findings. While directly relevant, the reported measurements of Kim *et al*.^25^ appear to contain inconsistencies, which makes it challenging to use them for comparison. For instance, it is not immediately clear why the contact radius changes linearly with time at a constant contact angle on a horizontal substrate. It is known that the square of the contact radius (or volume to the power of 2/3) scales linearly with time, not the radius itself. On the other hand, however, the approximate expression for the total rate of evaporation proposed by Sáenz *et al*.^24^ (based on their combined experimental-numerical examinations) offers unambiguous estimates against which our calculations can be contrasted. The suggested empirical formula relates *J* to the area and average curvature of the droplet’s free surface, denoted, respectively, by ${{\mathbb{S}}}_{d}$ and $\bar{H}$, as

36 $$J=2.24\,{{\mathbb{S}}}_{d}^{0.53}\,{\bar{H}}^{0.07}.$$

**Figure 8:**
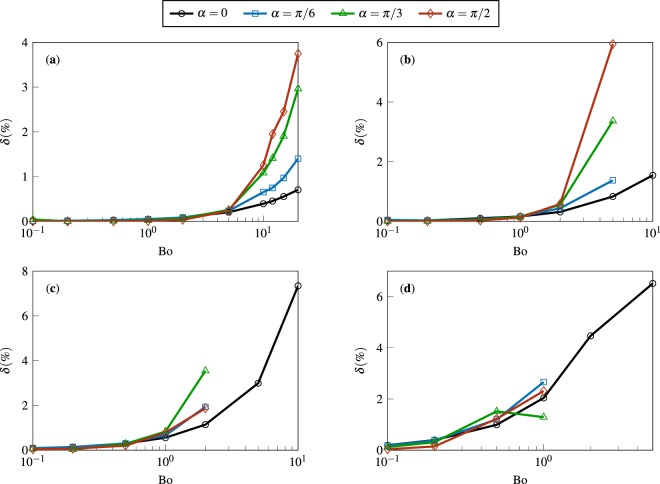
The percent difference *δ* between the results of Fig. 6 (for *J*/*J*^(0)^ versus Bo for various *α*) and the predictions of the scaling law proposed by Sáenz *et al*.^24^ (see Eq. (36)). Panels (a–d) correspond to ${\theta }_{c}=\pi /6,\,\pi /3,\,\pi /2,\,{\rm{and}}\,2\pi /3$, respectively.

Figure 8 presents the percent difference between our results for *J*/*J*^(0)^ (see Fig. 6) and the predictions of Eq. (36). The plots show excellent agreement between the *OpenFOAM* calculations and the conjectured scaling law.

## Summary

We have theoretically investigated the evaporation of a sessile droplet pinned on an inclined surface, tilted to an angle *α*. Starting by finding the droplet shape, we analytically solved the linearly-perturbed Young-Laplace equation to derive a first-order expression in terms of the Bond number for the profile of the drop. We also numerically calculated the drop geometry using an energy minimization method. This showed that our analytical and numerical models for the deformation of the droplet under the influence of gravity match very closely for a wide range of Bond numbers, well beyond the expected limit of ${\rm{Bo}}\ll 1$.

The total rate of evaporation, being the ultimate goal of our study, was calculated in a similar fashion to the droplet shape, i.e. both analytical and numerical approaches were used. Specifically, a perturbation theory coupled with a shortcut technique based on Green’s second identity were employed to obtain a first-order formula (again in term of Bo) for the evaporation rate, whereas a finite-volume method was utilized to carry out the numerical simulations. Perhaps surprisingly, we discovered that the rate of evaporation experiences only minor fluctuations as the droplet undergoes a sizable shape change driven by gravity. In addition, we found that the results of the perturbation theory, that only accounts for ${\mathscr{O}}(1)$ and ${\mathscr{O}}({\rm{Bo}})$ contributions, agree very well with those of simulations over an extensive span of Bo.

In conclusion, we note that the theoretical framework presented here can be extended for future studies on the evaporation-induced flow and particle deposition inside colloidal sessile drops sitting on titled substrates. For that purpose, the leading order correction to the vapor concentration field must be determined explicitly because the local mass flux is needed to fully determine the flow boundary conditions at the liquid-air interface (see, e.g., refs. ^49,50^). The outcome of such studies would shed light on the effect of gravity and substrate inclination angle on the so called coffee-ring phenomenon (see, e.g., refs. ^22,23^). Lastly, it is worth mentioning that our methodologies for calculating the droplet shape and its evaporation rate are equally applicable to two-dimensional drops, also known as cylindrical liquid lines (see, e.g., ref. ^51^).

## Supplementary information

Additional results for droplet shape
