# Publisher Correction: Evaporation of a sessile droplet on a slope

**DOI:** 10.1038/s41598-020-58811-z

**Published:** 2020-02-10

**Authors:** Mitchel L. Timm, Esmaeil Dehdashti, Amir Jarrahi Darban, Hassan Masoud

**Affiliations:** 1 Department of Mechanical Engineering-Engineering Mechanics, Michigan Technological University, Houghton, Michigan 49931 USA; 2 Department of Physics, University of Nevada, Reno, Nevada 89557 USA

Correction to: *Scientific Reports* 10.1038/s41598-019-55040-x, published online 24 December 2019

The HTML version of this Article was corrupted after publication leading to the incorrect display of equations. This has now been corrected; the PDF version of the paper was correct from the time of publication.

