# Supplementary material for: Evaporation of a sessile droplet on a slope
Source: Sci Rep. 2019 Dec 24;9:19803. doi: 10.1038/s41598-019-55040-x (PMC6930299; doi:10.1038/s41598-019-55040-x)
Supplement: Supplementary file 1 — Additional results for droplet shape [file 41598_2019_55040_MOESM1_ESM.pdf]

Evaporation of a sessile droplet on a slope

Mitchel L. Timm[1], Esmaeil Dehdashti[1], Amir Jarrahi Darban[2], and Hassan Masoud[1,*]

[1]Department of Mechanical Engineering-Engineering Mechanics, Michigan Technological University, Houghton, Michigan 49931, USA
[2]Department of Physics, University of Nevada, Reno, Nevada 89557, USA
[*]hmasoud@mtu.edu

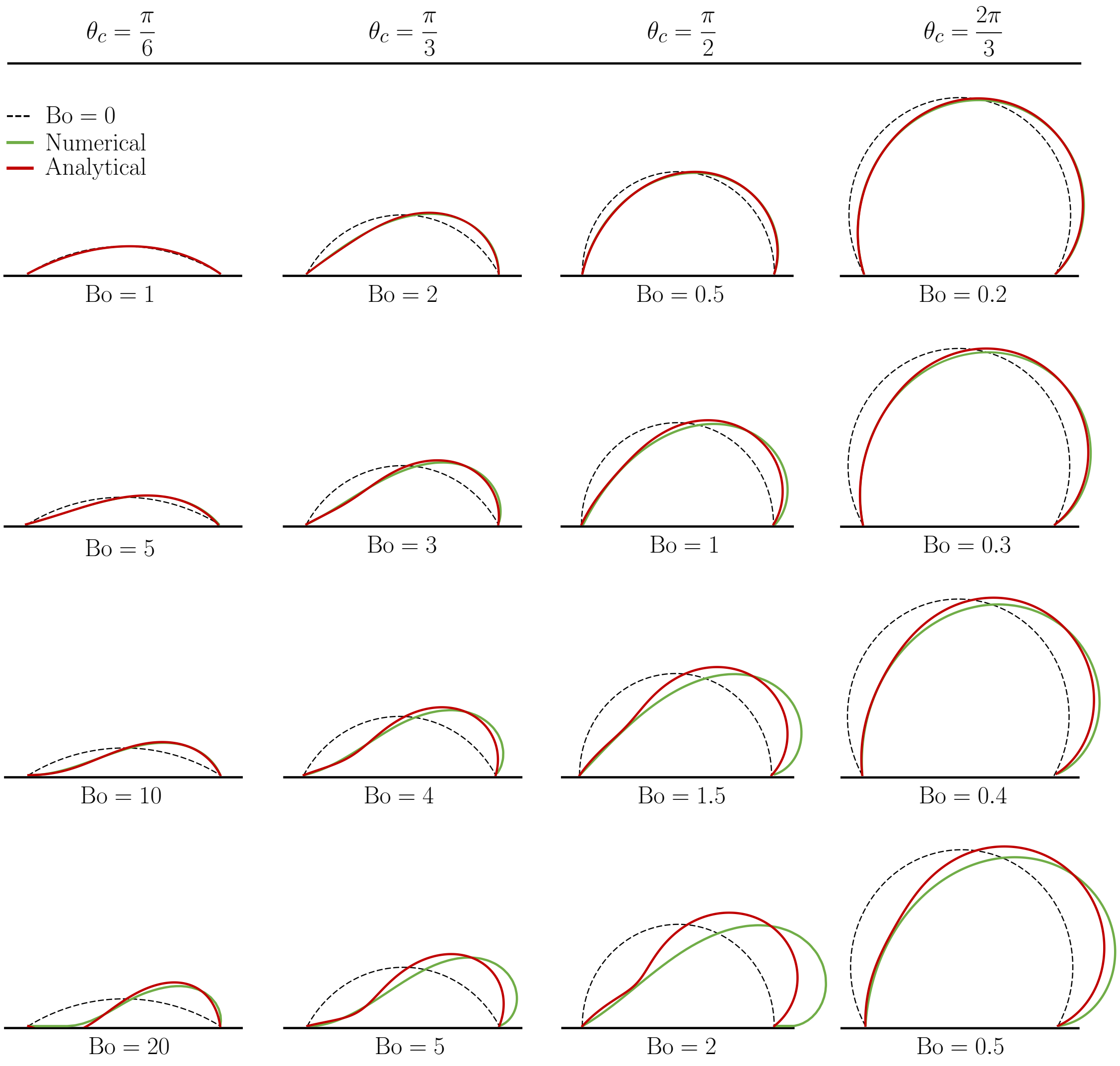

Figure 1S. Center-line profiles of sessile drops sitting on a tilted substrate with an inclination angle $\alpha = \pi/3$ for different values of Bo and θ_c.

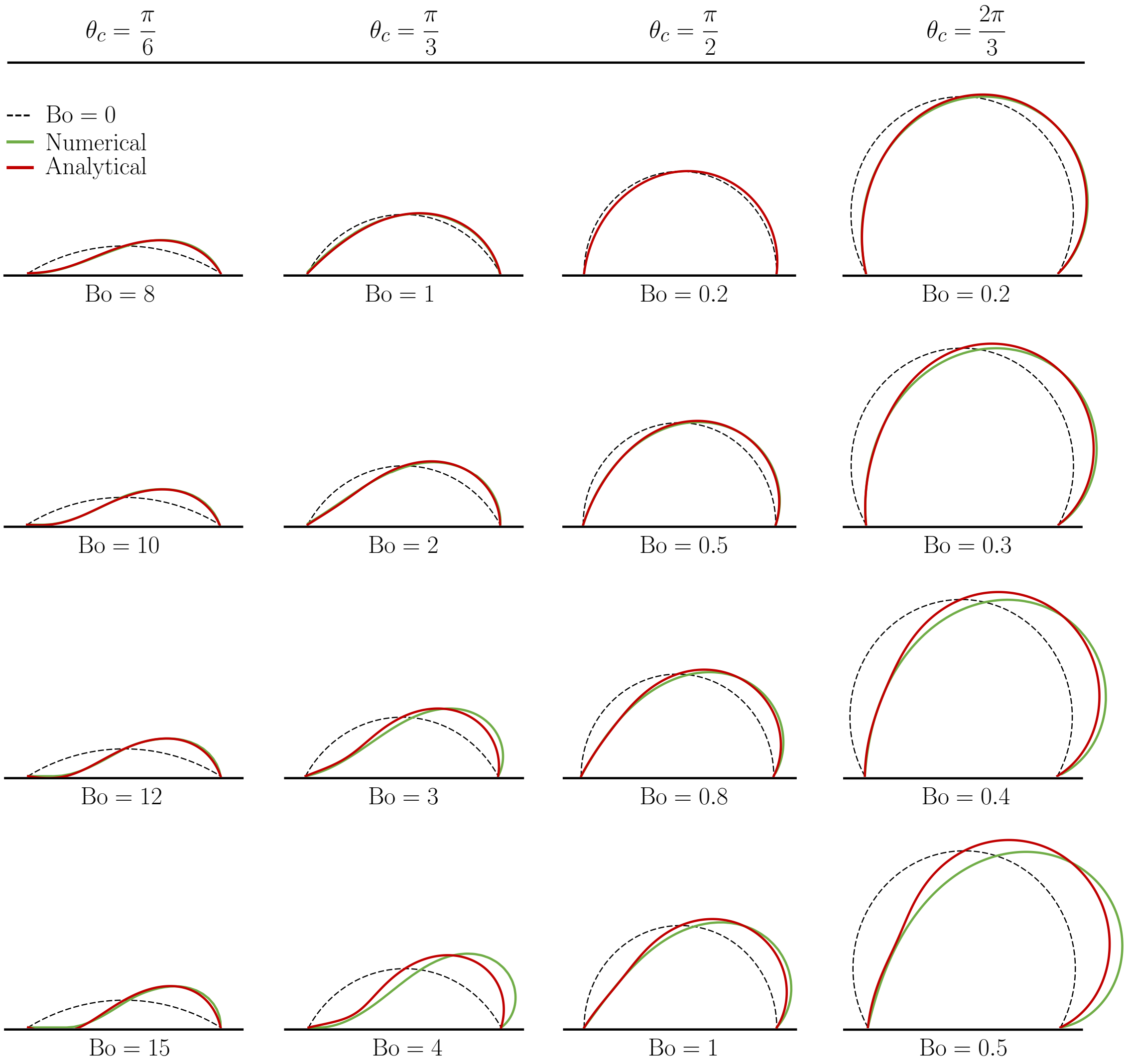

Figure 2S. Center-line profiles of sessile drops sitting on a tilted substrate with an inclination angle $\alpha = \pi/2$ for different values of Bo and θ_c.
